# Recent Advances in TiO_2_-Based Photocatalysts for Efficient Water Splitting to Hydrogen

**DOI:** 10.3390/nano15130984

**Published:** 2025-06-25

**Authors:** Muhammad Nisar, Niqab Khan, Muhammad I. Qadir, Zeban Shah

**Affiliations:** 1 Departamento de Ingeniería Eléctrica, Facultad de Ingeniería, Universidad Católica de la Santísima Concepción, Alonso de Ribera 2850, Concepción 4070129, Chile; 2 Centro de Energía, Universidad Católica de la Santísima Concepción, Alonso de Ribera 2850, Concepción 4070129, Chile; 3 São Carlos Institute of Physics, University of São Paulo (USP), P.O. Box 369, São Carlos 13560-970, SP, Brazil; 4 Instituto de Química, Universidade Federal de Goiás (UFG), Avenida Esperança s/n, Câmpus Samambaia, Goiânia 74690-900, GO, Brazil; 5 Institute of Chemistry, Federal University of Rio Grande do Sul, Av. Bento Gonçalves 9500, Porto Alegre 91501-970, RS, Brazil

**Keywords:** titanium dioxide (TiO_2_), solar energy, hydrogen production, doping and heterojunction

## Abstract

Titanium dioxide (TiO_2_) has been widely used as a potential candidate for the production of green hydrogen using the artificial photosynthesis approach. However, the wide bandgap (∼3.3 eV) of anatase TiO_2_ makes it difficult to absorb a large fraction of the solar radiation reaching the Earth, thus providing a low photocatalytic activity. Anatase TiO_2_ absorbs only 4% of solar radiation, which can be improved by engineering its bandgap to enhance absorption in the visible region. In the literature, many strategies have been adopted to improve the photocatalytic activity of TiO_2_, such as metal and non-metal doping and heterojunctions. These techniques have shown incredible enhancement in visible light absorption and improved photocatalytic activity due to their ability to lower the bandgap of pure TiO_2_ semiconductors. This review highlights different techniques like doping, heterojunctions, acidic modification, creating oxygen vacancies, and temperature- and pressure-dependence, which have improved the photochemical response of TiO_2_ by improving charge-transfer efficiencies. Additionally, the charge-transfer mechanism and enhancement in the photochemical response of TiO_2_ is discussed in each portion separately.

## 1. Introduction

Environmental pollution caused by the burning of fossil fuels has become an important challenge to tackle. The burning of fossil fuels leads to CO_2_ emissions, which consequently cause the greenhouse effect. The solar energy that strikes the Earth each hour is higher than all the energy consumed by humans in a year. The conversion of solar energy to another form can suppress these environmental issues caused by fossil fuels; however, it is a huge challenge for researchers to develop devices that utilize solar energy and convert it into other forms of energy [1]. The enormous demand for global energy is becoming a major concern for the scientific community. According to a 2013 report, the worldwide energy consumption was 17 terawatts (TW, equal to 10^12^ j/s) and will be double that by 2050, and subsequently triple by 2100 [2,3]. The increase in energy demand is significantly influenced by the growing global population, which is expected to reach approximately 9.9 billion by 2050, representing a 25% increase from the current 2023 population of 8.0 billion [4]. In addition, the increase in industrialization in recent years has also led to a high energy consumption and high demand for the Earth’s limited fossil fuel reserves which has increased the amount of CO_2_ discharge. The use of fossil fuels as an energy source has become one of the primary environmental issues of the 21st century. According to a report on climate change, CO_2_ emissions will increase to 590 ppm by the end of the 21st century. This excessive increase in CO_2_ levels could cause the Earth’s temperature to rise by up to 1.9 °C, leading to other damaging effects such as increased surface water levels and more frequent extreme weather events [5,6,7,8]. It is critical to find safe, Earth-abundant, sustainable, renewable, and clean energy resources to address these energy and environmental issues [9]. Several available renewable resources are safer, cleaner, contamination-free, and sustainable compared to fossil fuels, including wind, geothermal heat, and sunlight. However, each of these renewable energy resources has its restrictions; for instance, the expensive and harmful nature of hydropower generation from a dam, the difficulty of storing the electricity generated from wind, and the high operational cost of geothermal resources make it difficult to replace the currently used non-renewable energy resources [5]. In this scenario, hydrogen can be considered as an abundant and green renewable energy source that can solve the emission problems of dangerous greenhouse gases (CO_2_, CO, CH_4_), which are the primary source of global climate change [10]. The energy density of hydrogen is 143 MJkg^−1^, and this gas is a carbon-free energy carrier [11]. Using solar energy conversion to create clean and sustainable energy (green H_2_) by water splitting and transforming it to electric power through fuel cells is a key solution to worldwide energy concerns [3,12]. Additionally, the practical confines of the introduction of a cost-effective, energy-efficient photocatalytic system for water splitting is a major dispute that needs to be resolved for the successful achievement of a solar fuel-based economy [13].

Thus far, three important methods have been established for hydrogen production, comprising electrochemical, photochemical, and solar energy-assisted thermochemical methods [14]. The basis of the thermochemical technique is the application of concentrated solar radiation to act as a driving force to split water molecules; this is considered an important technology for ecological hydrogen production as a process in future energy structures [15]. Conceptually, the ideal simple process of “water thermolysis” comprises a one-step thermal decomposition of water, where solar energy is concentrated directly on chemical reactors to separate water into hydrogen and oxygen. However, the process has some limitations, as it requires an elevated temperature above 2500 K to ensure a realistic degree of decomposition and needs a good operational H_2_/O_2_ separation performance to hinder the recombination of these elements; thus, direct water splitting is not recommended [14]. Photocatalytic or photochemical water splitting is a process that utilizes light energy from the Sun to split water molecules into hydrogen and oxygen. In this process, a semiconductor serves as a photocatalyst, which in the presence of UV light or visible light splits water into pure oxygen and hydrogen. The photocatalyst absorbs photons and generates electron–hole pairs that drive the redox reaction. This sustainable method provides a green way to produce hydrogen, which can be further stored and converted to another form of energy [16,17]. Recently, several catalytically active semiconductors for photochemical reactions have been developed, but a stable material that shows worthy repeatability for visible light is yet to be introduced, and its discovery is still under investigation. Thus, for water splitting, it is imperative to develop suitable materials that can satisfy three main conditions: (i) show an outstanding stability in photochemical reactions; (ii) have a lower bandgap value than 3 eV; (iii) have an appropriate band edge potential for water splitting reactions [18].

**Figure 1:**
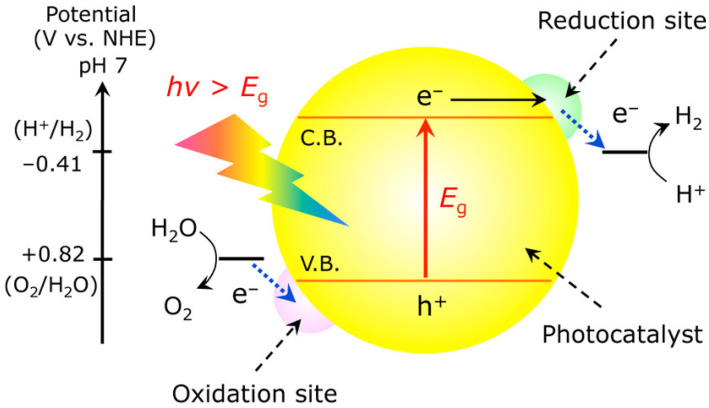
A typical model for photochemical water splitting, reproduced from Ref. [26], copyright 2013 American Chemical Society.

Since the pioneering discovery by Fujishima and Honda in the 1970s, numerous strategies have been developed to enhance the efficiency of photocatalytic hydrogen production [19]. In the last few decades, the exploration for the ideal materials to act as single photocatalysts has been a hot topic of research. Figure 1 illustrates a single photocatalytic system for water splitting using a semiconductor material. When irradiated with photons having energy equal to or greater than the bandgap, electrons in the valence band are excited to the conduction band, leaving behind positive holes in the valence band. The positive Gibbs free energy change (ΔG° = 237.13 kJ/mol) is a critical factor in overall water splitting, indicating that the reaction is non-spontaneous under standard conditions [20]. The strong reduction and oxidation potentials of photogenerated electrons and holes can drive redox reactions on the catalytic surface [21]. During the water splitting process, the formation of one molecule of H_2_ requires two electrons, whereas the formation of one molecule of O_2_ requires four holes. Therefore, the selection of a suitable semiconductor for effective water splitting primarily depends on the bandgap size, the position of the conduction band minimum (which must be more negative than the reduction potential), and the valence band maximum (which must be more positive than the oxidation potential). On the other hand, noble metals [22], enzymes, polyoxometalates (POMs), inorganic compounds, and organometallic complexes have been investigated as catalytic materials for electrochemical and photochemical H_2_ production from water. An especially interesting approach involves the use of metal–organic frameworks (MOFs) in aqueous solutions or in mixtures of water and organic solvents for photochemical water reduction and oxidation [23,24,25].

Different types of catalysts offer distinct advantages and disadvantages for chemical reactions. In the case of hydrogen production, both homogeneous and heterogeneous synthetic catalysts exhibit lower activity compared to hydrogenase enzymes. However, the practical use of hydrogenase is limited by its sensitivity to oxygen, high cultivation costs, and challenges in enzyme isolation, making it unsuitable for large-scale hydrogen production. To improve efficiency, noble metals such as platinum (Pt), palladium (Pd), and rhodium (Rh) have been loaded onto semiconductor materials. Although these metals enhance hydrogen production, their limited availability and high cost hinder their widespread use in large-scale photocatalytic fuel cells [27,28,29]. Considering factors such as simplicity, efficiency, cost-effectiveness, and environmental sustainability, photocatalytic water splitting is regarded as a promising method for hydrogen production. Generally, photocatalytic water splitting can be categorized into two main types: (a) photocatalytic (PC) or photochemical processes, and (b) photoelectrochemical (PEC) processes [30].

## 2. Photochemical Reaction

In photochemical reactions, light energy is directly utilized to initiate fundamental chemical transformations. This process typically relies on a powdered semiconductor photocatalyst that absorbs sunlight to generate electron–hole pairs, which subsequently drive the redox reactions involved in water splitting. Key factors influencing photocatalytic hydrogen production include the bandgap of the photocatalyst, surface area, efficiency of charge carrier separation, and stability under illumination. Studies have shown that, in photochemical systems, photocatalysts are commonly used as suspended particles within the reaction solution [30,31]. The authors highlighted several challenges in controlling key factors during the process, such as light absorption by suspended particles, pH fluctuations, and accurate determination of substrate concentration. These variables significantly influence the reaction kinetics, making the interpretation of experimental results difficult. Furthermore, conducting kinetic studies in suspension systems is often very time-consuming.

## 3. Basics of Photochemical Water Splitting

**Figure 2:**
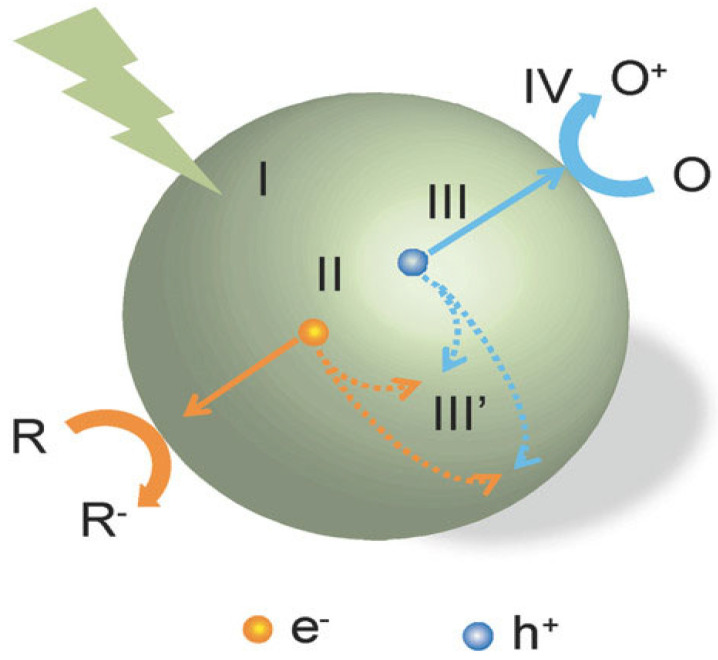
Steps involved in the photochemical reaction process [32].

The working principle of photochemical water splitting may be described completely in four main steps as illustrated in Figure 2: (I) absorption of incoming light to produce electron–hole pairs when its energy is equal or greater than the bandgap energy of the photocatalysts; (II) separation of excited electron–hole pairs; (III) transfer of electron–hole to the surface of photocatalysts; and (IV) redox surface reaction by these charges. During the third step, a significant portion of electron–hole pairs recombines on the surface site, releasing energy in the form of heat or light. Only the long-lived photogenerated charges have the potential to participate in the surface redox reactions, and their activity depends on the donor and accepter properties of the surface-adsorbed species [32].

## 4. Photoelectrochemical Reaction

The photoelectrochemical (PEC) reaction is a specialized form of photochemical reaction in which an external electric circuit facilitates the chemical reaction. In a PEC cell, a photocatalyst is deposited as a thin film on a conductive substrate to form a photoanode or photoelectrode, which is then immersed in an electrolyte solution to perform water splitting [30]. Hydrogen production in this system is enabled by the external circuit, which directs the photoinduced electrons from the photoanode to the photocathode. During water splitting, hydrogen and oxygen are produced simultaneously: the photogenerated electrons reduce protons at the photocathode to form hydrogen molecules, while oxygen is generated at the photoanode via water oxidation [33].

## 5. The Basic Concept Involves in PEC Water Splitting

Photoelectrochemical (PEC) water splitting is a complex process. Its fundamental principle involves applying an external bias to a photovoltaic or photoelectrode system immersed in an electrolyte containing redox couples. One of the electrodes is a semiconductor capable of absorbing light. Semiconductors have the unique ability to act as photocatalysts: when exposed to light, they generate electron–hole pairs that drive oxidation and reduction reactions. In PEC water splitting, the process of water electrolysis is assisted by both light and electricity. The photocatalytic electrodes absorb photons and provide the energy required for the redox reactions, while the externally applied electric or chemical bias supplies the additional voltage needed to overcome reaction barriers [34]. This external bias plays a crucial role in initiating and sustaining the reaction at a practical rate by overcoming the slow kinetics of charge transfer. Upon photon absorption, electrons are excited and electron–hole pairs are formed on the photoelectrode. The holes participate in oxidation reactions, while the excited electrons reduce protons (H^+^) to produce hydrogen gas (H_2_) [35,36].

On the other hand, several physical phenomena must be optimized in the design of an efficient device for photochemical water splitting, such as the interaction of light with matter. Three different phenomena—absorption, reflection, and transmission—can take place when light strikes a material’s surface. The extent to which light is absorbed or reflected depends on the properties of the medium; in media such as liquid electrolytes, only slight absorption or reflection typically occurs. Thus, minimizing the light propagation distance is recommended to reduce energy losses. Once the photon is absorbed inside the material, if its energy exceeds the bandgap of the semiconductor, it can excite an electron from the valence band to the conduction band, creating an electron–hole pair. The penetration depth—which refers to the depth at which photons are absorbed within the material—is an essential criterion for thin-film semiconductors.

The excitation of electrons to higher energy levels is accomplished through the absorption of photons. In a semiconductor, the overall distribution of electronic energy states arises from the individual atomic states, forming the valence band and the conduction band. The valence band edge (Ev) is the highest occupied energy level, and the conduction band edge (Ec) is the lowest unoccupied energy level; the difference between these two edges is called the bandgap (Eg). The classification of a semiconductor as n-type or p-type depends on the position of the Fermi level (the average energy of the electronic states) relative to the valence or conduction band. A p-type semiconductor is formed if the Fermi level is close to the valence band and the majority carriers are holes, whereas, if the majority carriers are electrons and the Fermi level is close to the conduction band, an n-type semiconductor is achieved.

One of the key phenomena, “charge separation and transport,” needs to be optimized for efficient material performance. The ultimate separation of light-induced electron–hole pairs is essential, as their premature recombination significantly limits the extraction of photocurrent. Thus, to generate a usable photovoltage and photocurrent, an external potential can be applied to drive the spontaneous separation of photogenerated charges. In designing a p-n junction, p-type and n-type semiconductors are brought into contact, and to reach equilibrium, free electrons and holes diffuse across the junction, resulting in a redistribution of charge. This results in the formation of a space–charge region, where a strong internal electric field is established. The electric field drives electrons and holes in opposite directions. High conductivity is crucial for efficient charge transport, and it depends on both carrier mobility and concentration.

Similarly, chemical reactions occur when photogenerated holes or electrons reach the surface of the photon-absorbing material. Water splitting involves either hydrogen or oxygen evolution reactions; therefore, in addition to charge separation and transport, the efficiency of the reaction and the mechanism of electron transfer from reactants to products is important. The role of the catalyst in accelerating the chemical reaction is vital. Electrolytes without significant depletion steadily support electrochemical reactions while the introduction of a catalyst lowers the activation energy by providing an alternative reaction pathway, often through the formation of intermediate species on the catalyst surface. Only a small amount of catalyst is required, as the reaction takes place on the surface [37]. Below are the equations describing the general photoelectrochemical water-splitting mechanism [34].

(1) 2H_2_O + hν → 2H_2_ + O_2 _ΔG° = 4.92 Ev (113 kcal m^−1^)

where hν is the photon energy

(2) 4H_2_ + 4e^−^ **→** 2H_2_ E°_red_ = 0 V

(3) 2H_2_O **→** 4H^+^ + 4e^−^ + O_2_ E°_red_ = 1.23V

## 6. Factors Affecting the Photochemical (PC) and Photoelectrochemical (PEC) Performance

**Figure 3:**
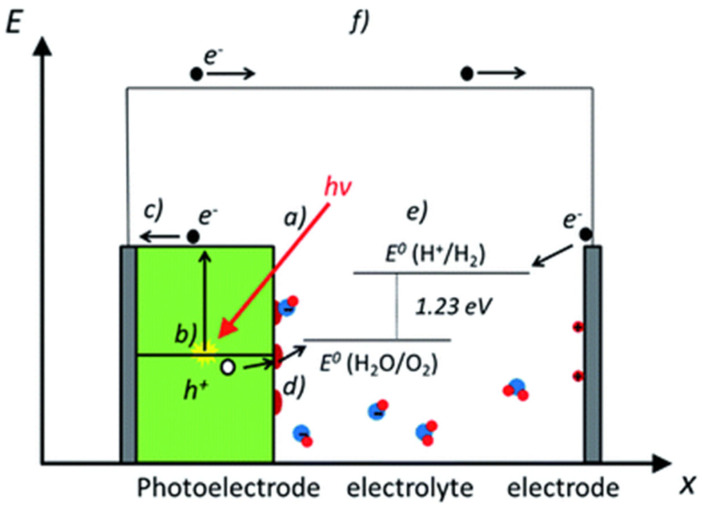
The scheme shows the basic phenomena involved in photoelectrochemical (PEC) water splitting: (a) interaction of mater with light, (b) generation of electron–hole pairs, (c) separation and transportation of charge (d), catalysis (e), water-splitting reaction (f), photocurrent associated with PEC water splitting [37]. Copyright 2020 Royal society of chemistry.

The photochemical and photoelectrochemical (PEC) water-splitting performance primarily depends on the morphology, uniformity, and structural order of the nanomaterials used. In addition, the size and structure of the materials are also important factors in the PEC water-splitting mechanism. However, PEC water splitting differs from the photochemical process in that it involves an external bias, and the excitation and de-excitation of electrons occur at separate electrodes. Furthermore, a PEC system requires a three-electrode setup—comprising a working electrode, counter electrode, and a reference electrode—to complete the reaction. To achieve efficient water splitting using these processes, several factors must be considered, including the structure and morphology of the material, the bandgap and suitable band edge positions for water oxidation and reduction, as well as pH, pressure, and temperature. The basic phenomena involves in the PEC water splitting is shown in (Figure 3).

### 6.1. Crystalline Structure and Morphology

**Figure 4:**
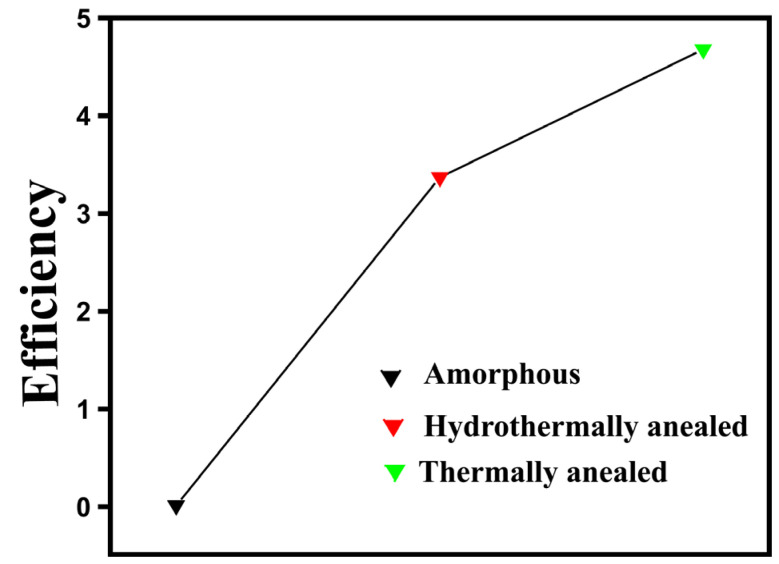
Photocurrent efficiency of different forms of TiO_2_ nanotubes [40].

The structure and morphology of photocatalysts generally depend on the synthesis method and the specific steps involved; for instance, variations in crystal size, shape, and structure can result from changes in temperature and the use of different surfactants. In addition, morphology and dimensionality are strongly influenced by pH. The nucleation and growth behavior can be controlled by the concentration of OH^−^ ions [38]. The performance of crystalline materials compared to amorphous materials is typically superior. A well-known example is the enhanced photocurrent observed in annealed (crystalline) TiO_2_ nanotubes compared to their amorphous counterparts. Figure 4 presents a comparison of the photodegradation performance of annealed and amorphous TiO_2_, showing that amorphous TiO_2_ can be transformed into crystalline TiO_2_ at elevated temperatures around 300 °C. As a general rule, crystalline TiO_2_ nanotubes demonstrate improved hydrogen production in PEC water splitting. Studies have shown that the crystallite size of TiO_2_ nanotubes increases with increasing voltage during anodization. This is likely due to the fact that higher voltages favor the formation of larger crystal nuclei and enhanced crystallinity. In turn, this reduces the number of defect sites that promote electron–hole recombination, thus, significantly affecting photocurrent efficiency [34,39,40].

### 6.2. Bandgap

**Figure 5:**
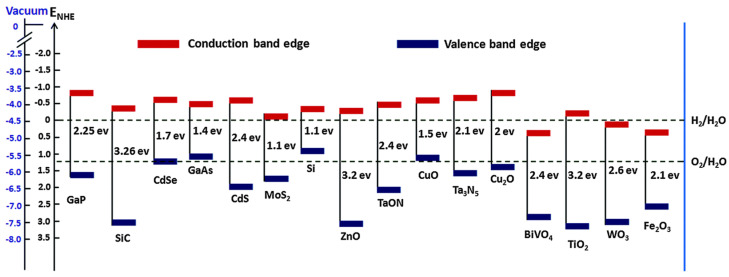
Schematic illustration of band position of various semiconductors vs. NHE and vacuum [44].

The electronic structure of semiconductors is typically described in terms of energy bands, which can be viewed as a range of energy resulting from the small energy differences between adjacent molecular orbitals [33]. For efficient PEC water splitting, bandgap energies in the range of 1.6 to 2.2 eV are essential. Semiconductors with large bandgaps are generally unable to absorb a significant portion of the visible light spectrum, resulting in reduced solar-to-hydrogen conversion efficiency [41]. The advantage of nanomaterials is their ability to modify bandgap energies to improve the PEC performance [42,43]. The bandgap can be reduced through the introduction of donor–acceptor species into the semiconductor. The band structure of various semiconductors in relation to the redox potential of water splitting is illustrated in Figure 5. Numerous strategies, including the modification of photocatalysts with transition metal cations, have been explored to improve solar energy utilization under visible light irradiation.

### 6.3. The Dependence on Temperature and Pressure

Temperature is an important parameter that plays a vital role in the performance of PEC cells. This is because catalytic activity, electrode kinetics, photoelectrode quantum efficiency, charge transfer, electrode stability, diffusion, and conductivity in the electrolyte, as well as ionic mobility, are all significantly influenced by temperature [45]. Typically, high temperatures are used in PEC experiments. However, improved PEC efficiency at low temperatures has also been reported. One study demonstrated a 95% increase in IPEC efficiency at 350 nm under low-temperature conditions [46]. Operating at low temperatures is particularly important, especially for fabricating PEC photoelectrodes from organic substrates with low melting points, and for studying interfacial physics to improve PEC performance. On the other hand, a study by Chong et al. [47] showed that temperatures above 80 °C can inhibit reactant adsorption, resulting in a reduced photocatalytic activity of TiO_2_. These findings indicate that the optimal operational temperature varies depending on the material. Therefore, the temperature factor can be adjusted to enhance the photocatalytic activity. Additionally, enhancement of the bandgap with increasing pressure has also been reported [48].

### 6.4. Effect of pH

Experimental results have shown that the band edge potential can be altered by changes in the pH of the electrolyte solution [31]. Additionally, the pH of the solution influences the charge state of the metal oxides. A study by Nada et al. [49] demonstrated that an acidic medium favors greater hydrogen production than a basic medium. More broadly, PEC cells operate under a wide range of pH conditions. The pH of the electrolyte solution significantly affects the equilibrium of the reaction, which determines the net total charge as either positive, zero, or negative at the surface. However, in highly corrosive or extreme pH environments, minimizing photocorrosion is essential to maintain efficiency. The electrode surface deteriorates as the movement of ions takes place during the reaction. The stability of the photoelectrode in varying pH conditions can be improved by the incorporation of nanomaterials. Similarly, photocatalysts show enhanced performance and stability in the presence of buffered electrolyte solutions [50]. Other factors influencing PEC water splitting include the intensity of incident light, dopant-induced effects on grain nucleation, impurities, surface facet exposure, water adsorption/desorption kinetics related to pore structure, and preparation-dependent grain morphology.

### 6.5. Limitations of Photochemical vs. Photoelectrochemical Approaches

Photochemical and photoelectrochemical approaches both offer promising pathways for green hydrogen (H_2_) production, each with their distinct advantages and limitations. Although the two processes differ fundamentally, the photochemical process operates without the need for an external bias and can utilize suspended catalysts in solution, making it relatively simple, cost-effective, and attractive for large-scale applications. However, the photochemical approach suffers from low quantum efficiencies and poor charge separation, which result in significant electron–hole recombination [51]. In contrast, the photoelectrochemical process enhances charge separation through the application of an external bias or by employing photoelectrodes within a cell. This improves conversion efficiencies and provides better control over reaction kinetics. Ultimately, the choice between photochemical and photoelectrochemical approaches depends on the specific target application. The photochemical approach favors simplicity and low cost, while the photoelectrochemical method offers higher efficiency and tunability at the expense of increased system complexity.

## 7. Titanium Dioxide (TiO_2_)

Over the years, TiO_2_ has gained significant attention and has become one of the most extensively studied photocatalysts due to several advantages, such as low cost, high photocatalytic efficiency, non-toxicity, thermal and chemical stability, and a high refractive index [52]. However, its photocatalytic performance is hindered by the rapid recombination of photogenerated electron–hole pairs, backward reactions, and its wide bandgap (3.02–3.18 eV), which limits its activity in the visible region. The wide bandgap of pristine TiO_2_ enables the absorption of only ultraviolet light (λ ≈ 380 nm), which constitutes just 4–5% of the solar spectrum, thereby reducing solar-to-chemical conversion efficiency. Furthermore, the fast recombination of electron–hole pairs lowers the quantum efficiency [53]. To address this, various metal oxides and sulfides have been developed and employed in hydrogen evolution reactions. However, many of these materials exhibit limited stability under different pH conditions, which is critical during photocatalytic reactions. For example, sulfide-based photocatalysts degrade under solar irradiation due to the oxidation of sulfide ions to elemental sulfur [54]. Metal oxides such as BiVO_4_, ZnO, WO_3_, CuO, Fe_2_O_3_, and others generally suffer from rapid charge recombination and various stability issues. Fe_2_O_3_-based photocatalysts have short hole diffusion lengths, leading to quick charge recombination under solar irradiation. BiVO_4_ is also prone to instability and fast charge recombination, and its band edge positions are suitable only for water oxidation [55,56,57]. Similarly, CuO is unstable due to rapid oxidation to Cu^2+^ and Cu^1+^ species [58].

On the other hand, TiO_2_ is stable, inexpensive, suitable for water redox reactions, and abundantly available, making it one of the most promising candidates for water-splitting applications [33,59]. Among various TiO_2_ nanomaterials, TiO_2_ nanotubes have emerged as a particularly attractive option. However, the previously mentioned limitations of TiO_2_ must be addressed to achieve higher efficiency. To overcome these challenges, numerous strategies have been developed. Notably, both metal and non-metal doping have been widely explored to enhance charge carrier separation and improve the optical absorption properties of TiO_2_. Other techniques include the formation of heterojunctions to facilitate charge separation and transport, surface modification through the deposition of noble metals or inorganic acids, metal ion incorporation, and dye sensitization [60]. Alternatively, black TiO_2_ nanoparticles have demonstrated superior photocatalytic activity compared to their pristine counterparts [61]. Additionally, TiO_2_ nanowires have shown a better performance than nanorods, indicating that the morphology and size of TiO_2_ nanostructures are critical factors influencing conversion efficiency [36,62]. Liu et al. [40] reported an enhancement in cell performance when using thermally annealed TiO_2_ nanotubes, attributed to improved charge transport due to increased crystallinity. Scanning electron microscopy (SEM) cross-sectional images revealed that, prior to annealing, the nanotubes had smooth inner and outer wall morphology. Thermal annealing induced roughening of the inner tube walls, while the diameter and outer wall remained largely unchanged. In contrast, water-annealed nanotubes showed pronounced morphological changes. The current–voltage (*I–V*) curves presented in the study indicated that water-annealed nanotubes exhibited approximately threefold higher dye adsorption compared to amorphous nanotubes. However, thermally annealed nanotubes achieved a superior solar cell performance, with a solar conversion efficiency of approximately ƞ~4.65%.

### Role of TiO_2_ in HER

TiO_2_ is a promising material for the hydrogen evolution reaction (HER) due to its unique electronic and physicochemical properties. Its wide bandgap provides strong oxidative stability and excellent resistance to photocorrosion, which is crucial for long-term operation in aqueous environments. However, the intrinsic HER activity of pristine TiO_2_ is limited by its poor electrical conductivity and sluggish charge-transfer kinetics. These limitations can be effectively addressed through strategies such as metal or non-metal doping, defect engineering (e.g., introducing oxygen vacancies), and the formation of heterojunctions with conductive cocatalysts such as Pt, MoS_2_, or graphene. These modifications introduce mid-gap states, enhance charge separation, and improve electron mobility. Importantly, the conduction band edge of TiO_2_ lies at a sufficiently negative potential to thermodynamically favor proton reduction to H_2_ under illumination, enabling efficient solar-driven HER. Furthermore, its tunable surface chemistry and high surface area in nanostructured forms provide abundant active sites and improve mass transport, making TiO_2_ a highly versatile platform for the development of efficient and stable HER systems, particularly in photoelectrochemical cells.

## 8. TiO_2_ Modification Strategies

### 8.1. Doping of Metal and Non-Metal

As a general rule, the functionality and efficiency of nanomaterials are significantly influenced by their lattice structure and surface composition. Literature reports indicate that modifying the crystal lattice of nanomaterials by introducing electronically active secondary species can markedly alter charge carrier dynamics and optical absorption properties. The deposition of certain metals facilitates the attraction of electrons to the metal particles deposited on TiO_2_, thereby reducing electron–hole recombination and consequently enhancing photocatalytic activity [63]. The primary role of metallic dopants is to introduce additional energy levels within the bandgap, which shifts the optical absorption edge by lowering the energy barrier [64]. Among noble metals, Ag-modified TiO_2_ is widely studied for hydrogen generation under low-energy UV illumination. Liu et al. [65] synthesized TiO_2_ nanotubes decorated with Ag_2_S nanoparticles, which were subsequently treated with acetonitrile and subjected to vacuum drying to obtain Ag_2_S-decorated TiO_2_ nanotubes with a narrower bandgap. Their results demonstrated a hydrogen production rate of 1.13 mL/cm^2^/h, with excellent reproducibility. Similarly, Lalitha et al. [66] reported the impregnation of Ag^+^ ions onto the TiO_2_ surface, attributing the observed hydrogen evolution under solar irradiation to the interface formed by Ag ions on the TiO_2_ surface layers. Among the various synthesis techniques, the sol–gel method is considered one of the simplest and most commonly used methods for nanoparticles synthesis. Its advantages include rapid processing, good compositional homogeneity, and operation at relatively low calcination temperatures and times, often resulting in nanoparticles with a high crystallinity and surface area [52].

Dholan et al. [67] employed a combination of room-temperature (RT) and sol–gel methods to synthesize Fe- and Cr-doped TiO_2_. The photocatalytic performance of the doped TiO_2_ was evaluated for H_2_ production under visible light irradiation. The doped samples exhibited enhanced hydrogen evolution compared to undoped TiO_2_, attributed to the role of Fe and Cr as electron trapping agents. In another study, Wu and Lee demonstrated that the photocatalytic H_2_ generation from pure water and/or water containing sacrificial reagents significantly increased upon deposition of Pd, Ni, and Pt onto TiO_2_. Additionally, enhanced hydrogen production using TiO_2_ loaded with Cu metal in aqueous methanol solution has also been reported [68]. Zhu et al. [69] deposited Pt nanoparticles on hollow-sphere TiO_2_ via a sol–gel technique for hydrogen generation. The inclusion of approximately 1.0 wt% Pt resulted in the highest H_2_ evolution rate. This improvement was attributed to the surface dispersion of Pt nanoparticles, which effectively act as charge separators. Ismail et al. [63] reported the incorporation of PdO into a mesoporous TiO_2_ network, leading to increased photocatalytic performance. They observed catalytic activity 4- and 2-times higher than that of commercial TiO_2_-P_25_ and Pd/TiO_2_-P_25_, respectively. Huang et al. [70] applied a combined hydrothermal/sol–gel technique to synthesize co-doped TiO_2_ nanorods with Rh and Nb. These nanorods were tested under visible and UV light irradiation for hydrogen production. Among the samples, Ti_0_._996_Nb_0_._002_Rh_0_._002_O_2_ exhibited superior photocatalytic activity compared to pure TiO_2_ and other co-doped materials. The enhanced performance was attributed to improved charge separation and the introduction of Rh, which creates impurity levels above the valence band of TiO_2_.

**Figure 6:**
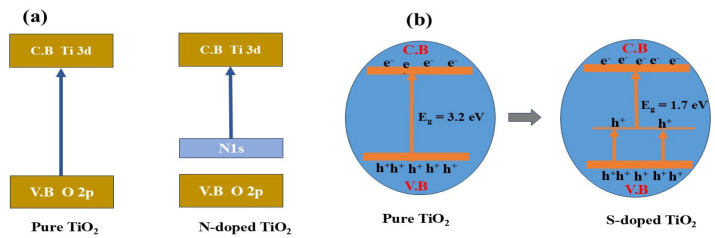
Schematic diagram of the energy band structure of pure TiO_2_ and N-doped TiO_2_ (**a**), and sulfur (S) doping narrowing the bandgap energy (**b**).

Recent studies confirm that metal ion doping (cations) initiates a deep localization of d-states within the bandgap of TiO_2_, thereby promoting charge carrier recombination. In contrast, anionic dopants are less prone to forming recombination centers and are generally more effective in enhancing photocatalytic activity [71]. Various anionic dopants, such as N, C, F, B, S, and Cl, have been extensively investigated for TiO_2_ modification. Hou and co-workers [72] synthesized nitrogen (N)-doped TiO_2_ nanotubes using a hydrothermal method. The resulting materials were immersed in deionized water and ammonia solutions of varying concentrations. Their findings showed that the sample treated with ammonia solution exhibited higher light absorption compared to that treated with water alone. They also observed that the morphology of TiO_2_ was strongly influenced by the molar ratio between deionized water and ammonia solution. Moreover, nitrogen doping reduced the bandgap energy from 3.23 to 2.84 eV (Figure 6a), thereby significantly enhancing the visible-light activity of the N-doped TiO_2_ nanotubes. More recently, McMananmon et al. [73] studied sulfur (S)-doped TiO_2_ nanoparticles prepared via sol–gel synthesis. Their study revealed that sulfur atoms occupied interstitial sites within the TiO_2_ crystal lattice, leading to the formation of surface states between the valence band (VB) and conduction band (CB). The bandgap energy was red-shifted from 3.2 to 1.7 eV, facilitating visible-light absorption as shown in Figure 6b. In 2004, Luo and co-workers [74] reported the synthesis of Br and Cl co-doped TiO_2_ using a hydrothermal technique. Titanium chloride and hydrobromic acid served as sources of Ti and Br, respectively. The resulting material exhibited enhanced absorption in the visible region due to non-metal doping, which contributed to bandgap narrowing and improved solar-driven water-splitting efficiency.

**Figure 7:**
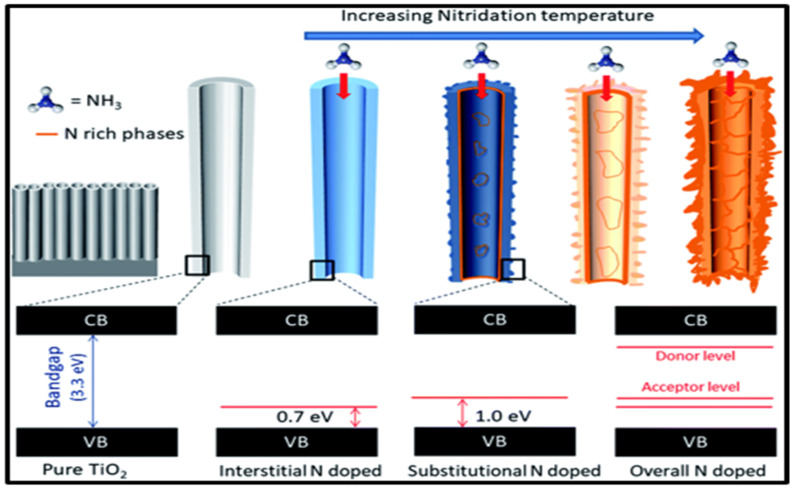
Schematic representation of N doping and the presence of interband states related to interstitial and substitutional doping. Adopted from Reference [75].

In 2021, Khan et al. investigated the effects of both substitutional and interstitial nitrogen (N) doping on the bandgap of TiO_2_. The researchers employed a straightforward nitridation method to synthesize N-doped TiO_2_ nanotubes (NTs). N-doping in TiO_2_ results in the formation of donor states below the conduction band and acceptor states above the valence band, which collectively contribute to a reduced bandgap and enhanced visible light absorption, as illustrated in Figure 7 [75].

**Figure 8:**
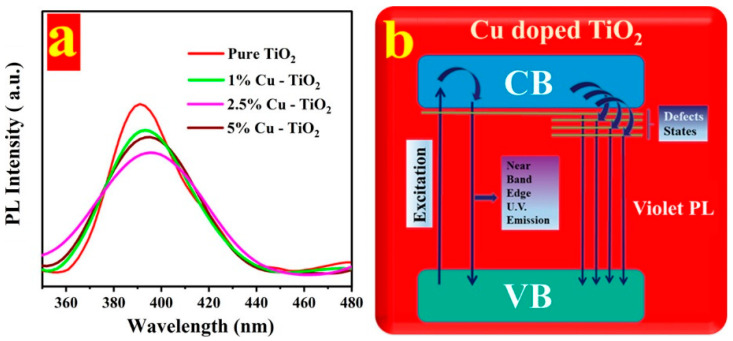
(**a**) PL spectra of Cu-doped TiO_2_ and (**b**) scheme explaining the possible transitions. Adopted from reference [76].

Recently, Fazil et al. synthesized copper (Cu)-doped TiO_2_ nanoparticles (NPs) via hydrothermal synthesis. Doping levels of 1%, 2.5%, and 5% Cu were applied to TiO_2_, resulting in a noticeable redshift in bandgap energies. Specifically, the bandgap of pure TiO_2_ redshifted from 3.19 eV to 3.8 eV for the 2.5% Cu-doped sample. This shift was attributed to the formation of impurity states or vacancies between the valence band (VB) and conduction band (CB) induced by the dopants. Additionally, the authors hypothesized that a reduced bandgap could affect the photocatalyst’s morphology, particle size, electronic, and optical properties [76]. Photoluminescence spectra, presented in Figure 8, demonstrated enhanced charge transfer and a reduced bandgap for the 2.5% Cu-doped TiO_2_ NPs, leading to improved overall photochemical activity.

Most recently, Safinaz et al. synthesized pure TiO_2_ loaded with metals (Co, Ni, and Rh) as cocatalysts using a hydrothermal synthesis procedure [77]. They observed a significant change in the bandgap energies upon loading these metallic cocatalysts. The bandgap of pure TiO_2_ was found to be 2.97 eV, which redshifted to 2.6 eV after cocatalyst loading. The authors did not specify the exact cause of this bandgap reduction. However, they attributed the decrease to heterojunction formation between TiO_2_ and the cocatalysts, which enhanced the photocatalytic performance. Although doping is widely used to tailor the properties of TiO_2_, it also introduces several drawbacks. It can create defects such as vacancies and interstitials, which serve as charge recombination centers, thereby reducing photocatalytic efficiency. Excessive doping may also impair crystallinity and induce undesirable phase transitions—for instance, promoting the anatase-to-rutile transformation. Moreover, improper doping can introduce deep impurity states within the bandgap, leading to charge trapping and recombination [78,79].

### 8.2. Creation of Heterojunction

**Figure 9:**
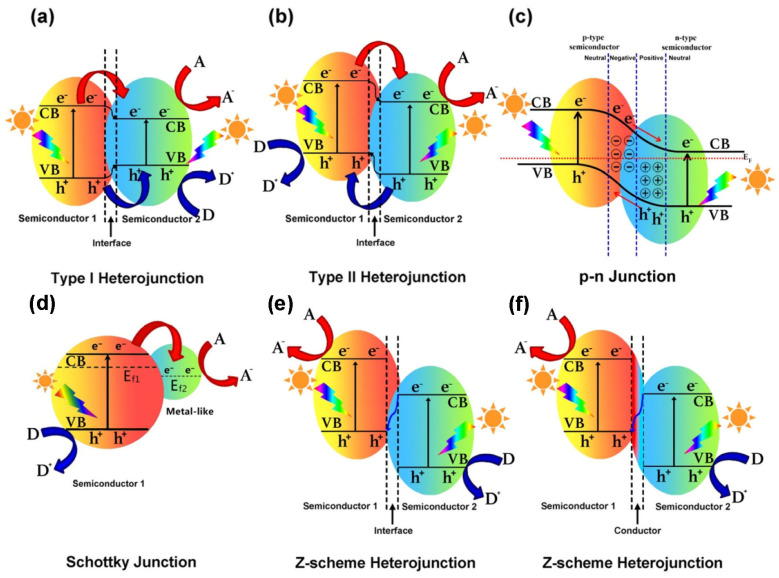
Various types of heterojunctions and their respective band structure: (**a**) type-I hetero junction, (**b**) type-II heterojunction, (**c**) p-n junction or heterojunction, (**d**) Schottky junction, (**e**) and (**f**) Z-scheme junctions. A, D, and E_F_ denote electron accepter, electron donor, and Fermi level, respectively [89]. Copyright 2019 Elsevier Ltd.

Global energy demand and the pursuit of environmentally friendly energy sources have been a critical issue in recent years. The transformation of solar energy into a storable energy medium is highly desirable [80]. While a single semiconductor can achieve direct water splitting, its effectiveness depends on two key criteria: appropriate redox potentials and strong absorption in the visible light region [81]. In general, suitable semiconductor photocatalysts must possess a wide bandgap with well-aligned valence band (VB) and conduction band (CB) positions, efficient separation of photoinduced charge carriers, high resistance to photocorrosion, and the ability to transfer energetic electrons. Although TiO_2_ meets most of these requirements, its major limitation is that it can only absorb UV light, which constitutes approximately 4% of the solar spectrum [82]. A heterojunction is typically defined as the interface between two dissimilar semiconductors with mismatched band structures. Introducing heterojunctions—by coupling TiO_2_ with other semiconductors or noble metals that have wider or narrower bandgaps—has been widely explored to overcome this limitation. The construction of such heterojunctions can significantly enhance light absorption, suppress the recombination of charge carriers, and produce a more stable photocatalyst [26]. Therefore, selecting a suitable cocatalyst is a critical and competitive step. A photocatalyst with a large surface area provides abundant active sites, and accurate band alignment is essential for efficient charge transfer between the two semiconductors [2,83]. Based on these considerations, heterojunctions are commonly classified into five types, as illustrated in Figure 9.

#### 8.2.1. Type I Heterojunction

In a type-I heterojunction, the valence band (VB) of semiconductor 1 lies below and the conduction band (CB) of semiconductor 1 lies above those of semiconductor 2, as shown in Figure 9a. Therefore, under light illumination, both the photogenerated electrons and holes migrate from semiconductor 1 to semiconductor 2.

#### 8.2.2. Type II Heterojunction

The type-II heterojunctions have a staggered band structure between the two applied semiconductors, which effectively inhibits the recombination of photoinduced electron–hole pairs. Semiconductors 1 and 2 possess distinct band potentials, as presented in Figure 9b. Upon light illumination, the transfer of a photogenerated hole in the type-II junction can easily take place from the valence band (VB) of semiconductor 2 to the VB of semiconductor 1, provided that the VB of semiconductor 2 is lower than that of semiconductor 1. Conversely, if the conduction band (CB) of semiconductor 1 is higher than the CB of semiconductor 2, the transfer of electrons from semiconductor 1 to semiconductor 2 takes place, causing the spatial separation of charge carriers. This leads to an increase in the electron lifetime and reduces electron–hole recombination. Furthermore, when sufficient energy is supplied to excite semiconductor 1, semiconductor 2 acts as an electron–hole acceptor. Notably, the construction of type-II heterojunctions is highly promising for photocatalytic applications. Hence, the type-II heterojunction is considered one of the best heterojunction-based photocatalysts, according to the literature [84,85,86,87,88].

#### 8.2.3. p-n Heterojunction

The improvement of electron–hole pair separation in other heterojunction types is often insufficient to effectively suppress electron–hole recombination in semiconductors. In this context, the idea of assembling a p-n heterojunction is presented to boost photocatalytic performance by providing a built-in electric field to speed up charge carrier transfer. The p-n heterojunction exhibits higher charge separation efficiency than the type-II heterojunction, thereby enhancing photocatalytic activity. The p-n heterojunction is obtained by combining p-type and n-type semiconductor materials [90,91,92,93,94,95]. As the Fermi level varies depending on the semiconductor type, the Fermi level of the p-type semiconductor is located closer to the valence band (VB), while that of the n-type is closer to the conduction band (CB). Therefore, electron–hole diffusion occurs between the semiconductors even before light illumination. As soon as the Fermi level reach equilibrium, the circulation of charge carriers stabilizes [96]. The creation of a built-in electric field due to different Fermi-level positions in semiconductors is responsible for the separation of electron–hole pairs, as shown in Figure 9c.

**Figure 10:**
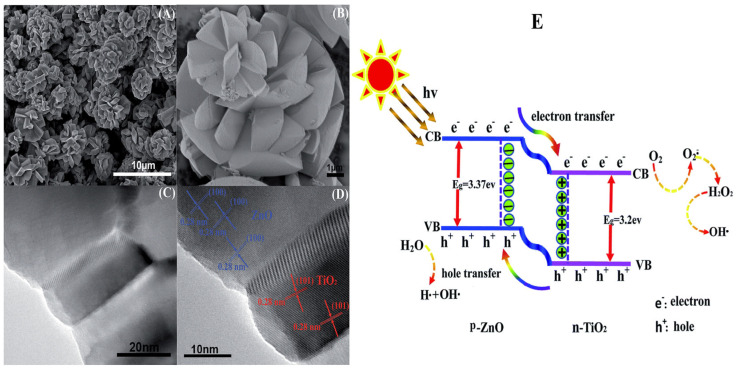
SEM, TEM, and HRTEM images of fan blades (**A**–**D**), respectively. Schematic diagram illustration of p-n heterojunction (**E**) [97]. Copyright 2015, Royal Society of Chemistry (RCS).

Zha et al. [97] demonstrated the photocatalytic performance of a TiO_2_/ZnO nanocomposite under UV light for methyl orange degradation. Their results showed an excellent photocatalytic activity (97% in 30 min), attributed to its large surface area and hierarchical structure. The SEM and TEM images (Figure 10A, Figure 10B, and Figure 10C, respectively) of the as-prepared heterojunction reveal a multi-layered, fan-bladed structure with an average length ranging between 2.5 and 3 µm. Figure 10D presents the HRTEM image, where the planes (101) and (100) correspond to TiO_2_ and ZnO, respectively. Figure 10E shows the photocatalytic mechanism of the TiO_2_/ZnO heterojunction. The ZnO energy bandgap (3.37 eV) is slightly greater than that of TiO_2_, thus presenting a more negative conduction band (CB) and valence band (VB) potentials. Upon UV illumination, photons excite electrons from the VB to the CB in both semiconductors. Due to the conduction band offset, electrons preferentially migrate to the CB of TiO_2_. Meanwhile, holes thermodynamically transfer from the VB of TiO_2_ to the VB of ZnO, enhancing the photocatalytic activity.

**Figure 11:**
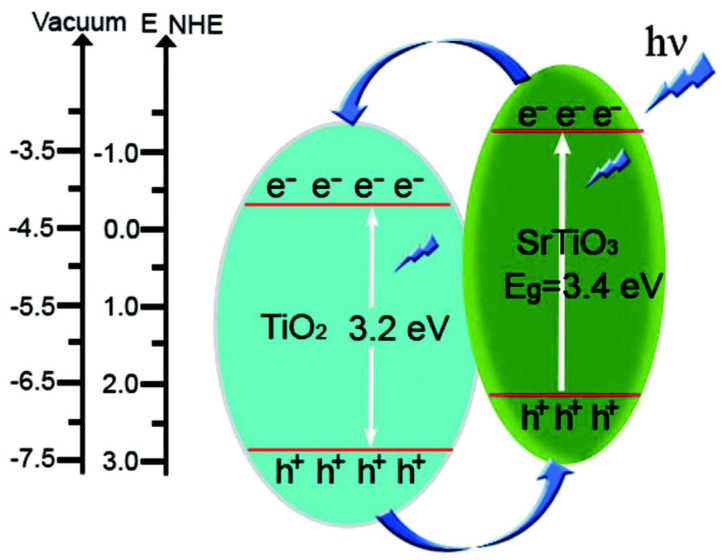
Scheme showing the bandgap energy and charge transfer or separation pathways in SrTiO_3_/TiO_2_ composite [98]. Copyright 2015, Royal Society of Chemistry (RSC).

Guo et al. [98] reported the successful synthesis of SrTiO_3_/TiO_2_ nanosheets using a hydrothermal process, which resulted in a porous structure and a high surface area. An enhancement in charge carrier separation was observed under UV light irradiation. The photoinduced charge transfer and separation in the SrTiO_3_/TiO_2_ composite are illustrated in Figure 11.

**Figure 12:**
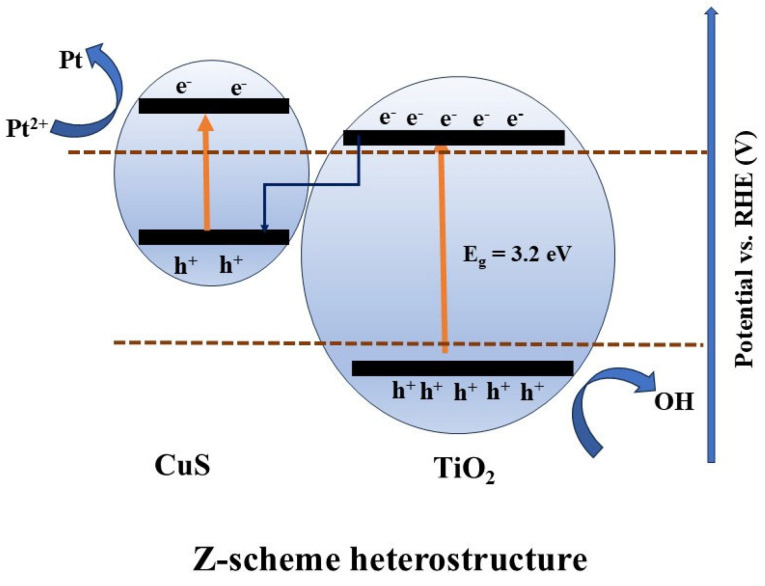
Scheme representing the charge transfer in CuS/TiO_2_.

In another study, Liu et al. [99] constructed a CuS/TiO_2_ heterojunction using solvothermal synthesis, exhibiting a spherical, cake-like shape. The resulting material exhibited suppressed charge recombination and efficient charge transfer, attributed to the well-established built-in electric field formed at the CuS/TiO_2_ p-n heterojunction. In addition, the heterojunction enhanced light absorption due to a narrowed bandgap, thereby improving photocatalytic activity. The charge-transfer mechanism in CuS/TiO_2_ is shown in Figure 12.

Recently, Dongxiang et al. [100] carried out density functional theory (DFT) and nonadiabatic molecular dynamics (NAMD) analysis on a TiO_2_-based heterojunction. In this study, they formed a heterojunction between anatase TiO_2_ (A-TiO_2_) and rutile TiO_2_ (R-TiO_2_). The study suggested that the A-TiO_2_/R-TiO_2_ heterojunction effectively prolongs the lifetime of photogenerated electron–hole pairs and improves the redox capability. Furthermore, the A-TiO_2_/R-TiO_2_ heterojunction demonstrates a higher solar-to-hydrogen (STH) efficiency under varying pH conditions.

#### 8.2.4. Schottky Junction

The heterojunction between a semiconductor and a metal-like material is called a Schottky junction, which is highly effective at generating a space–charge separation region. Due to its favorable interfacial structure, electrons rapidly transfer from one component to the other, facilitating Fermi level alignment and significantly hindering charge recombination, thereby improving photocatalytic performance [89]. Copper (Cu), cobalt (Co), and nickel (Ni) are considered potential candidates in photocatalysis because of their high work function. Combining these cocatalysts with TiO_2_ can form a Schottky junction at the interface, resulting in a built-in electric field, or can create active sites, both of which enhance electron–hole separation and thereby improve the photocatalytic activity of pristine TiO_2_ [101].

Qiu et al. [102] constructed a Cu/TiO_2_ Schottky junction using the solvothermal synthesis procedure. They used different percentages of Cu with TiO_2_, with optimal charge separation observed for the 10% Cu–TiO_2_ junction. The enhancement in charge separation was attributed to the formation of the built-in electric field. This built-in electric field further facilitates charge separation, thereby enhancing photocatalytic hydrogen production.

**Figure 13:**
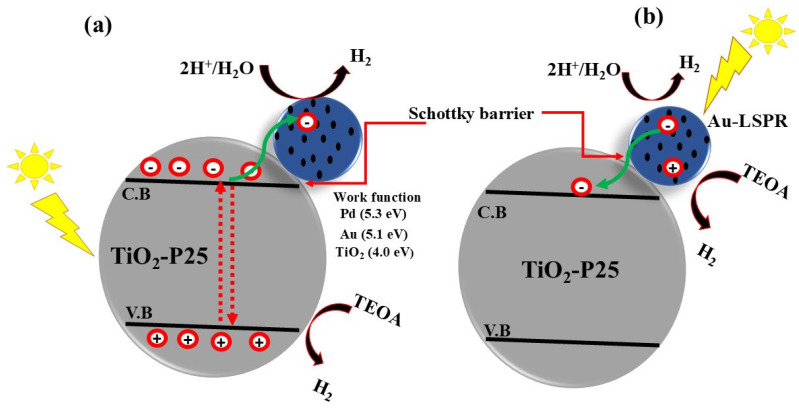
Scheme representing photocatalytic mechanism under (**a**) UV, and (**b**) visible light irradiation. The corresponding work function and Schottky barrier formation of the Au, Pd, and TiO_2_ are also given. Adopted from references.

Recently, Medrano et al. [103] modified commercial TiO_2_ with mono- and bimetallic (Au, Pd, and AuPd) nanoparticles using the chemical reduction method. This study reported that Au, Pd, and AuPd, in combination with TiO_2_, form a Schottky barrier that enhances electron–hole separation and photocatalytic H_2_ production. The Schottky junction is formed by the high-work-function metals Au, Pd, and AuPd with TiO_2_, which creates an internal electric field. This internal field facilitates effective charge separation and inhibits electron–hole recombination, thereby enhancing photocatalytic activity. The schematic diagram for the AuPd-loaded TiO_2_ is shown in Figure 13, illustrating the photocatalytic mechanism under UV and visible light irradiation.

#### 8.2.5. Z-Scheme Heterojunction

All the earlier-mentioned heterojunctions can effectively increase charge separation in photocatalysts. However, the redox capacity of the semiconductor composite materials is often compromised, since the reduction and oxidation reactions typically occur on the semiconductor with the less favorable redox potentials [104]. In 1979, Brad et al. [105] reported the Z-scheme photocatalytic method for the first time to suppress the above-mentioned issue. Inspired by the natural photosynthesis process in plants, the Z-scheme enables both effective photogenerated charge carrier separation and the preservation of strong redox capability.

However, limited application of the Z-scheme system using a redox mediator in the liquid phase has been noted [106]. The development of an all-solid-state photocatalytic Z-scheme heterojunction system is highly desirable [107], which leads to the proposal of a solid conductive material that acts as an electron mediator—or even operates in the absence of a mediator (Figure 9e,f). Nanoscale metals possess excellent electrical conductivity, which makes them ideal candidates as electron mediators in the construction of Z-scheme systems [108,109,110]. In contrast, Yu et al. [111] in 2013 advanced the concept of a mediator-free Z-scheme heterojunction. In the direct Z-scheme charge-transfer mechanism, photogenerated electrons transfer from the conduction band (CB) of semiconductor 2 to the valence band (VB) of semiconductor 1, where they recombine with the photogenerated holes. Unlike the traditional liquid-phase Z-scheme, the all-solid-state Z-scheme system can be widely applied in both gas–solid and liquid–solid photocatalytic systems [107].

**Figure 14:**
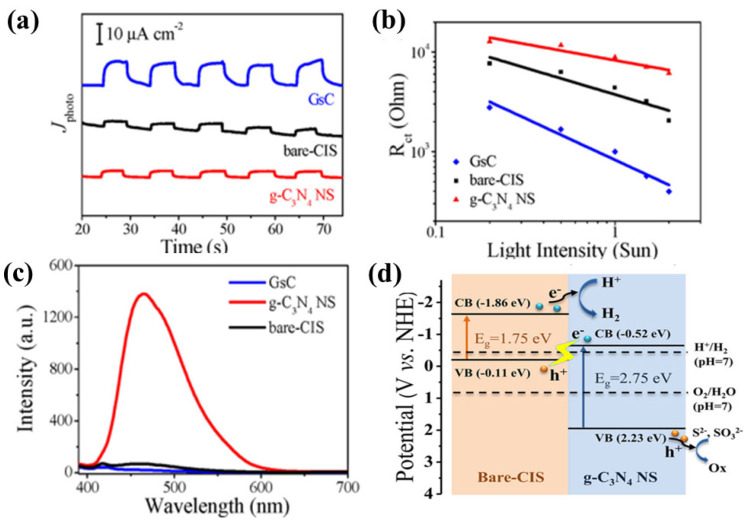
Transient photocurrent of the samples at λ > 420 nm (**a**), the effect of the light intensities on the R_et_ (**b**), PL spectra of the samples (**c**), band potential of the materials used for designing a Z-scheme system (**d**) [116]. Copyright 2018 American Chemical Society.

As mentioned above, constructing an indirect Z-scheme by introducing an electron mediator between the two semiconductors can enhance charge transfer. In contrast, a direct Z-scheme system improves photocatalytic activity by enabling a tight interface between the two components [112,113,114,115]. In this context, Li et al. [116] reported a direct Z-scheme photocatalyst composed of g-C_3_N_4_ nanosheets (g-C_3_N_4_ NS) and CuInS_2_ (denoted as GsC). Their results showed that GsC exhibited a significantly higher photocurrent density compared to neat CuInS_2_ and g-C_3_N_4_ NS, as illustrated in Figure 14a, indicating efficient charge separation. Figure 14b further confirms the lowest charge-transfer resistance (R_et_) of GsC across all light intensities when compared to bare CuInS_2_ and g-C_3_N_4_ NS, suggesting easier electron migration in GsC toward the electrolyte protons, facilitating H_2_ generation. Remarkably, the photoluminescence (PL) lifetime of GsC showed a 6-fold enhancement, as clearly depicted in Figure 14c, demonstrating superior performance in separating photogenerated electron–hole pairs (Figure 14d).

In 2021, Lv et al. [117] established a mesoporous Cu_2_O/TiO_2_ Z-scheme system for photocatalytic hydrogen evolution using the solvothermal synthesis method. The study suggested that the combination of TiO_2_ with Cu_2_O improved both stability and charge carrier mobility. Moreover, the Cu_2_O/TiO_2_ composite enhances the number of active sites, which are crucial for the rapid migration of hydrogen ions (H^+^) and the generation of hydrogen gas (H_2_). The Cu_2_O/TiO_2_ Z-scheme system retains the strong reduction potential of the conduction band (CB) of Cu_2_O and the oxidation potential of the valence band (VB) of TiO_2_, resulting in improved photocatalytic performance.

Recently, Liu et al. [99] constructed a CuS/TiO_2_ Z-scheme heterojunction via the co-precipitation synthesis method. The study reported that Z-scheme construction using CuS and TiO_2_ improves charge transfer due to the difference in carrier concentrations between the two semiconductors. Electrons diffuse from TiO_2_ to CuS, while holes move from CuS to TiO_2_. This bidirectional charge diffusion between the semiconductors leads to the formation of a built-in electric field, which is the primary driving force for enhanced charge separation and transport to oxidation and reduction sites, thereby improving photocatalytic activity.

It is clear from the above discussion that Z-scheme and type-II heterojunctions are comparable in terms of energy band structure but follow different charge-transfer mechanisms. In this regard, how to differentiate between the two types of heterojunctions has become a hot topic in scientific studies. The presence of a mediator in the heterojunction indicates a Z-scheme system in a hybrid photocatalyst. However, in the absence of a mediator, the possibility of both type-II and Z-scheme mechanisms exists. Therefore, more accurate strategies are required to investigate the charge-transfer mechanism. Ohno et al. [118] used dual-beam photoacoustic (DB-PA) spectroscopy to confirm the Z-scheme pathway in the WO_3_/g-C_3_N_4_ system, in comparison to a type-II heterojunction. In addition, the detection of reactive oxygen species such as superoxide (O_2_^−^) and hydroxyl (•OH) radicals relative to redox potentials using chemical probe techniques can also be proposed as a strategy to verify the Z-scheme mechanism.

The TiO_2_ heterojunction with other metal oxides is widely employed to enhance photocatalytic performance. However, it has several shortcomings. Poor band alignment and interfacial defects can promote electron–hole recombination instead of facilitating efficient charge separation. Many materials are used to form heterojunctions (for example, CdS, MoS_2_) with TiO_2_, but they often suffer from photochemical instability, leading to performance degradation over time [119]. Moreover, the fabrication of high-quality, defect-free heterojunctions is complex and difficult to scale for industrial applications. Furthermore, lattice mismatches and environmental concerns regarding certain materials, such as toxic heavy metals, pose additional challenges to the practical deployment of TiO_2_-based heterojunctions.

### 8.3. Inorganic Acid Modification of TiO_2_

The use of inorganic acids as surface modifiers for TiO_2_ photocatalysts can significantly alter their surface charge carrier properties in neutral water. The addition of new surface groups to TiO_2_ can greatly facilitate the separation of photogenerated electron–hole pairs, thereby improving photocatalytic activity. As a general rule, surface modification with inorganic acids enhances O_2_ adsorption on the photocatalyst surface, leading to superior photocatalytic performance [120]. Cui et al. [121] reported the surface modification of TiO_2_ with phosphate. The results showed better photocatalytic activity for phosphate-modified TiO_2_ than for unmodified TiO_2_. They proposed that phosphate modification does not affect the crystal phase composition, optical properties, or crystallinity. Hence, the enhancement in O_2_ adsorption is primarily related to the linkage of phosphate groups on the TiO_2_ surface. Phosphate-modified TiO_2_ alters the surface terminal groups from –Ti–OH to –Ti–O–P–OH groups [122]. In fact, the –Ti–O–P–OH group is more acidic than the –Ti–OH group, as the dissociation of –Ti–O–P–OH in water occurs to some extent, producing –Ti–O–P–O^−^ and hydrogen ions (H^+^). Thus, increased surface acidity is expected to favor O_2_ adsorption. This interpretation is supported by the temperature-programmed desorption of ammonia (NH_3_-TPD) curves for treated and untreated TiO_2_, and further corroborated by FT-IR spectra of adsorbed pyridine. Additionally, Cui et al. [121] modified TiO_2_ with various inorganic acids, including nitric acid, sulfuric acid, and phosphoric acid. After thermal treatment at 500 °C, HNO_3_ was removed from the modified TiO_2_ due to its instability, whereas H_3_PO_4_ and H_2_SO_4_ remained as residues on the TiO_2_ surface. Generally, the greater the amount of chemically adsorbed O_2_, the stronger the surface photovoltage spectroscopy (SPS) intensities. The SPS intensities followed the order of TiO_2_ < TiO_2_/HNO_3_ < TiO_2_/H_2_SO_4_ < TiO_2_/H_3_PO_4_, confirming that TiO_2_ treated with H_2_SO_4_ and H_3_PO_4_ had a larger amount of chemically adsorbed O_2_. The photocatalytic activity of TiO_2_ treated with H_3_PO_4_ and H_2_SO_4_ was clearly higher than that of the untreated samples. These experimental results indicate that TiO_2_ surface modification is a key strategy for enhancing its photocatalytic activity.

### 8.4. Modification of TiO_2_ by Creating Oxygen Vacancies

A scientifically important and interesting type of TiO_2_ modification involves the creation of oxygen vacancies by removing oxygen atoms. Reduced TiO_2_ is generally represented as TiO_2−x_, where x denotes the estimated density of oxygen vacancies. The Magnéli phase, a titanium suboxide with the general formula Ti_n_O_2n−1_, is formed by removing a small percentage of oxygen and has attracted significant attention due to its potential to enhance the photocatalytic activity of TiO_2_. Magnéli-phase titanium suboxide was first produced in the 1950s using hydrogenation [71]. The photocatalytic hydrogen evolution performance of metal- and non-metal-doped TiO_2_, as well as heterojunctions (e.g., Z-scheme, S-scheme, etc.) involving TiO_2_ and other semiconductors, is summarized in Table 1. The hydrogen evolution rates are listed in a dedicated column, while the reaction parameters are provided separately. Moreover, to enable a better comparison of the photochemical and photoelectrochemical performance of TiO_2_-based photocatalysts, the photocurrent densities of TiO_2_-based photoelectrodes are also included in Table 2.

## 9. Conclusions and Future Direction

In summary, TiO_2_-based photocatalysts and their application in the production of green energy (H_2_) have been discussed. Strategies such as doping, heterojunction formation, acidic modification, oxygen vacancy engineering, and the influence of temperature and pressure have been discussed to improve charge separation. Metal and non-metal doping in TiO_2_ reduces the bandgap and improves charge separation by enhancing visible light absorption. Likewise, forming a heterojunction between TiO_2_ and another semiconductor improves charge separation by forming a built-in electric field at the interface. Acidic modification favors enhanced hydrogen production compared to basic media because it modifies the band edge potential. Temperature and pressure also affect the photocatalytic activity of TiO_2_. For instance, temperatures above 80 °C can hinder reactant adsorption, while increased pressure can widen the bandgap, thereby suppressing the photocatalytic activity of TiO_2_. In addition, a comparative table has been constructed to summarize the photocatalytic hydrogen production performance of pure TiO_2_ and its combinations with other semiconductors, metals, and non-metals. To conclude, heterojunction formation and doping are the most promising and effective strategies for enhancing charge separation and transfer efficiency in TiO_2_ by engineering the bandgap and creating a built-in electric field.

This review provides critical insights into bandgap tuning, charge carrier dynamics, and morphology-dependent effects, which offer practical guidance for the rational design of next-generation TiO_2_-based photocatalysts. Furthermore, it highlights the synergistic effects of combining multiple modification strategies, helping to overcome challenges in optimizing TiO_2_ for scalable and sustainable hydrogen production.

Finally, considering the inherent limitations of TiO_2_, future research should focus on developing new catalysts with improved performance via scalable and cost-effective synthesis routes. The synthesis process should strategically incorporate metallic dopants to effectively engineer the bandgap, thereby enhancing light absorption and charge separation. This should be complemented by the controlled deposition of cocatalysts to further boost catalytic activity and stability. Additionally, advancements in in situ characterization techniques and computational modeling will play a pivotal role in optimizing charge dynamics and defect engineering at the atomic level. The integration of these strategies is expected to accelerate the development of highly efficient and durable systems, ultimately enabling the practical implementation of solar-driven hydrogen production technologies.

## Figures and Tables

**Table 1:** Photocatalytic (photochemical) hydrogen evolution for TiO_2_ doped with metals and non-metals, and making it a heterojunction with other semiconductors under different reaction conditions.

Entry	Catalysts	H_2_ Production	Parameters	Ref
1	Co, Ni, and Cu-doped TiO_2_	85 µmol h^−1^	UV irradiation Methanol	[123]
2	Ga or Ni co-doped TiO_2_	35 µmol h^−1^	125 W Hg lamp ≥ 400 nm Methanol	[124]
3	Cu and S-TiO_2_	7.5 mmol g^−1^ h^−1^	500 W Xe lamp, using quartz reactor	[125]
4	Ni-doped TiO_2_	180 µmol h^−1^	300 W Hg lamp methanol	[126]
5	Bi-doped TiO_2_	4500 µmol g^−1^ h^−1^	500 W, using Xe lamp	[127]
6	N-doped TiO_2_	102 µmol g^−1^ h^−1^	Visible light irradiations	[128]
7	B-N-TiO_2_	85 µmol/50 mg methanol	36 W light lamp	[129]
8	S-doped TiO_2_	164 mmol g^−1^ h^−1^	Visible light (1.5 G) irradiation ≥ 400 nm	[130]
9	Nb-doped TiO_2_	0.7 mmol g^−1^ h^−1^	Hg(UV)-Xe (Vis) lamp (500 W)	[131]
10	TiO_2_-SiO_2_	1.7 cm^3^/h.gcat	300 W Xe lamp, visible light irradiation (λ > 420 nm)	[132]
11	Cu_3_P/TiO_2_ Z-scheme	607 µmol g^−1^ h^−1^	300 W Xe lamp Water, methanol	[133]
12	Znln_2_S_4_/ TiO_2_ Z-scheme	6.5 mmol g^−1^ h^−1^	300 W Xe lamp irradiation TEOA	[134]
13	NiO-TiO_2_ p-n heterojunction	228 µmol g^−1^ h^−1^	300 W Xe lamp AM 1.5 G filter Water, methanol	[135]
14	Ru-doped TiO_2_	23.9 mmol g^−1^ h^−1^	UV-LED lamp, methanol	[77]
15	g-C_3_N_4_/ TiO_2_ S-scheme	134 µmol g^−1^ h^−1^	300 W Xe lamp TEOA	[136]

**Table 2:** Photoelectrochemical (PEC) performance of different TiO_2_-based heterojunctions.

Entry	Photoelectrode	Photocurrent Density	Parameters	References
1	Pure TiO_2_ and N-TiO_2_	180 µA/cm_2_	<0.3 V vs. Ag/AgCl, AM 1.5 G, 1 Sun illumination, and 1 M KOH	[75]
2	TiO_2_-BiFeO_3_/rGO	1.7 mA/cm^2^	0.6 V vs. Ag/AgCl, AM 1.5 G, 1 Sun illumination, 0.1 M NaOH	[100]
3	TiO_2_/BiVO_4_	2.2 mA/cm^2^	at 1.23 Vs RHE, 1 Sun illumination, AM 1.5 G	[137]
4	BiVO_4_/TiO_2_ NFs	1.7 mA/cm^2^	at 1.23 V vs. RHE, AM 1.5 G illumination, 1 M Na_2_SO_3_	[138]
5	R-TiO_2_@BiVO_4_	2.1 mA/cm^2^	0.45 V vs. RHE, AM 1.5 G illumination.	[139]
6	TiO_2_@CoNi-LDHs	4.4 mA/cm^2^	1.23 V vs. RHE, AM 1.5 G illumination, 1 M Na_2_SO_3_, pH 6.8	[140]
7	TiO_2_%40CoNi-LDHsTiO_2_@c/FeTiO_3_	6.03 mA/cm^2^	1.5 V vs. SCE	[141]
8	ZnS/CdS/TiO_2_	7.45 mA/cm^2^	0.25 M S/1 M Na_2_S	[142]
9	TiO_2_ NPs	100.12 μA cm^−2^	1.23 V vs. RHE, 0.5 M Na_2_SO_4_, 1.5 G illumination	[143]
